# Infant vaccinations and risk of childhood acute lymphoblastic leukaemia in the USA

**DOI:** 10.1038/sj.bjc.6690668

**Published:** 1999-09

**Authors:** F D Groves, G Gridley, S Wacholder, X O Shu, L L Robison, J P Neglia, M S Linet

**Affiliations:** Division of Cancer Epidemiology and Genetics, National Cancer Institute, Bethesda, Maryland, 20892-7244, USA; Department of Pediatrics, University of Minnesota Medical School, Minneapolis, USA

**Keywords:** acute lymphoblastic leukaemia, *Haemophilus influenzae*, vaccination, children

## Abstract

Previous studies have suggested that infant vaccinations may reduce the risk of subsequent childhood leukaemia. Vaccination histories were compared in 439 children (ages 0–14) diagnosed with acute lymphoblastic leukaemia (ALL) in nine Midwestern and Mid-Atlantic states (USA) between 1 January 1989 and 30 June 1993 and 439 controls selected by random-digit dialing and individually matched to cases on age, race and telephone exchange. Among matched pairs, similar proportions of cases and controls had received at least one dose of oral poliovirus (98%), diphtheria–tetanus–pertussis (97%), and measles–mumps–rubella (90%) vaccines. Only 47% of cases and 53% of controls had received any *Haemophilus influenzae* type b (Hib) vaccine (relative risk (RR) = 0.73; 95% confidence interval (CI) 0.50–1.06). Although similar proportions of cases (12%) and controls (11%) received the polysaccharide Hib vaccine (RR = 1.13; 95% CI 0.64–1.98), more controls (41%) than cases (35%) received the conjugate Hib vaccine (RR = 0.57; 95% CI 0.36–0.89). Although we found no relationship between most infant vaccinations and subsequent risk of childhood ALL, our findings suggest that infants receiving the conjugate Hib vaccine may be at reduced risk of subsequent childhood acute lymphoblastic leukemia. Further studies are needed to confirm this association and, if confirmed, to elucidate the underlying mechanism. © 1999 Cancer Research Campaign


*British Journal of Cancer* (1999) **81**(1), 175–178
© 1999 Cancer Research Campaign
Article no. bjoc.1999.0668

### Infant vaccinations and risk of childhood acute lymphoblastic leukaemia in the USA

**FD Groves[1], G Gridley[1], S Wacholder[1], XO Shu[2], LL Robison[2], JP Neglia[2] and MS Linet[1]**

[1]Division of Cancer Epidemiology and Genetics, National Cancer Institute, Bethesda, Maryland 20892-7244, USA, [2]Department of Pediatrics, University of Minnesota Medical School, Minneapolis, Minnesota, USA

**Summary** Previous studies have suggested that infant vaccinations may reduce the risk of subsequent childhood leukaemia. Vaccination histories were compared in 439 children (ages 0–14) diagnosed with acute lymphoblastic leukaemia (ALL) in nine Midwestern and Mid-Atlantic states (USA) between 1 January 1989 and 30 June 1993 and 439 controls selected by random-digit dialing and individually matched to cases on age, race and telephone exchange. Among matched pairs, similar proportions of cases and controls had received at least one dose of oral poliovirus (98%), diphtheria–tetanus–pertussis (97%), and measles–mumps–rubella (90%) vaccines. Only 47% of cases and 53% of controls had received any *Haemophilus influenzae* type b (Hib) vaccine (relative risk (RR) = 0.73; 95% confidence interval (CI) 0.50–1.06). Although similar proportions of cases (12%) and controls (11%) received the polysaccharide Hib vaccine (RR = 1.13; 95% CI 0.64–1.98), more controls (41%) than cases (35%) received the conjugate Hib vaccine (RR = 0.57; 95% CI 0.36–0.89). Although we found no relationship between most infant vaccinations and subsequent risk of childhood ALL, our findings suggest that infants receiving the conjugate Hib vaccine may be at reduced risk of subsequent childhood acute lymphoblastic leukemia. Further studies are needed to confirm this association and, if confirmed, to elucidate the underlying mechanism.

**Keywords**: acute lymphoblastic leukaemia; *Haemophilus influenzae*; vaccination; children

The possible association of infant vaccinations with risk of childhood leukaemia has been of interest for more than 30 years. In a small hospital-based case-control study of childhood leukaemia in Australia, an excess of diphtheria, tetanus and pertussis (DTP) vaccinations was observed among the cases (Innis, 1965). Subsequent studies, however, did not confirm this finding. The large, population-based Oxford Survey of Childhood Cancer reported protective effects from smallpox, diphtheria, tetanus, pertussis, measles, rubella, poliomyelitis and bacille Calmette-Guérin (BCG) vaccines among 5636 children with all forms of leukaemia (Kneale et al, 1986). Similarly, a case-control study of British children with all leukaemias reported a significantly reduced risk of leukaemia among children who received tetanus, diphtheria, pertussis, smallpox, polio and/or measles vaccines (McKinney et al, 1987). Also, a small case-control study of non-T-cell acute lymphoblastic leukaemia (ALL) in Japan reported protective effects for both BCG and measles vaccination (Nishi and Miyake, 1989), whereas a study of children with all leukaemia types in Germany reported a protective effect of unspecified vaccinations (Kaatsch et al, 1996).

We carried out a large case-control investigation of potential risk factors for childhood ALL conducted collaboratively by the National Cancer Institute (NCI) and the Children's Cancer Group (CCG) among children diagnosed prior to age 15. To investigate the influence of vaccination on ALL risk, we evaluated recorded information for all vaccines recommended for routine use in infancy among 439 ALL cases and an equal number of matched controls in nine Midwestern and Mid-Atlantic states in the USA.

*Received 24 November 1998*
*Revised 8 February 1999*
*Accepted 9 February 1999*

*Correspondence to:* FD Groves, Descriptive Studies Section, Biostatistics Branch, Epidemiology and Biostatistics Program, Division of Cancer Epidemiology and Genetics, National Cancer Institute, Executive Plaza – South, Room #8089, 6120 Executive Boulevard, MSC #7244, Bethesda, MD 20892-7244, USA

### METHODS

Patients with ALL aged 0–14, diagnosed between 1989 and 1993, were enrolled in the study (Kleinerman et al, 1997). Subjects who resided in Illinois, Indiana, Iowa, Michigan, Minnesota, New Jersey, Ohio, Pennsylvania, or Wisconsin at the time of diagnosis were eligible for the vaccination component of the study. Controls selected through random-digit dialing (Robison and Daigle, 1984) were individually matched to the cases by age (within 25% of the corresponding case's age at diagnosis), the first eight digits of the telephone number, and race (African-American/white/other). There were three separate components of this study, as described earlier (Kleinerman et al, 1997). Mothers of 96% of eligible cases and 75% of eligible controls from the nine states completed an initial telephone interview, and mothers of 788 (98%) and 699 (97%) eligible cases and controls, respectively, participated in a subsequent in-person interview. In the third component, vaccination data were provided by mothers (based on vaccination records from physicians) or obtained directly from the physicians of 630 (79.9%) cases and 550 (78.7%) controls, representing overall participation rates of 70.1% for cases and 50.4% for controls. There were 439 matched pairs for whom vaccination data were collected.

Data collection forms, completed by the mothers using vaccination records from physicians or by the physicians themselves, inquired whether and when the child had received oral or injected

**Table 1** Characteristics of cases and controls (439 pairs)

| | Cases | Controls |
|---|---|---|
| Male | 234 (53%) | 248 (56%) |
| Born | | |
| 1974–1984 | 142 (32%) | 139 (32%) |
| 1985–1987 | 175 (40%) | 174 (40%) |
| 1988–1992 | 122 (28%) | 126 (29%) |
| Age at censoring | | |
| < 6 months | 119 (27%) | 119 (27%) |
| 36–60 months | 144 (33%) | 144 (33%) |
| > 60 months | 176 (40%) | 176 (40%) |
| Day-care (ever) | 137 (31%) | 135 (31%) |
| Preschool (ever) | 162 (37%) | 177 (40%) |
| Family income | | |
| < $20 000 | 91 (23%) | 66 (17%) |
| $20 000–$29 999 | 118 (30%) | 101 (25%) |
| $30 000–$39 999 | 84 (21%) | 91 (23%) |
| $40 000+ | 105 (26%) | 140 (35%) |
| Mother's education | | |
| High School graduate or less | 184 (42%) | 168 (38%) |
| Some post-High-School education | 145 (33%) | 139 (32%) |
| College graduate | 110 (25%) | 132 (30%) |
| Father's education | | |
| High School graduate or less | 187 (43%) | 148 (34%) |
| Some post-High-School education | 126 (29%) | 137 (31%) |
| College graduate | 126 (29%) | 154 (35%) |

poliovirus vaccine, trivalent diphtheria–tetanus–pertussis vaccine, bivalent diphtheria–tetanus vaccine, bivalent tetanus–diphtheria vaccine, monovalent tetanus vaccine, trivalent measles–mumps–rubella vaccine, *Haemophilus influenzae* group b (Hib) vaccines, hepatitis B virus vaccine and other vaccines. For 329 (75%) of the cases and 334 (76%) of the controls among the 439 matched pairs, the mother provided vaccination records, and the physician was not contacted. With the mother's permission, a medical-record request was filed with the subject's primary physician, requesting details and dates of vaccination for the remaining subjects.

Vaccination data were sought but not obtained from 158 of the 788 cases whose mothers were interviewed in-person, of whom 63 were due to maternal refusal, and 94 were due to physician refusal or non-response. Likewise, 84 maternal refusals and 63 physician refusals or non-responses were the main reasons for the 149 controls without vaccination data among the 699 controls whose mothers were interviewed in-person. Vaccination data were coded up to the date of diagnosis for cases, and up to the date of interview for controls.

To ensure that the case and control within each pair were observed for the same length of time, we censored the vaccination data by adopting a common 'cut-off-age' (the lesser of the age at diagnosis for the case or the age at interview for the control) for both members of each matched pair, excluding from analysis any vaccine doses received after the cut-off age. We calculated relative risks and 95% confidence intervals for ALL following exposure to each vaccine (ever vs never vaccinated). We also evaluated number of doses of vaccine received, age at first dose, age at last dose, time between first dose of each vaccine and diagnosis, and time between last dose of each vaccine and diagnosis. Since none of these analyses contributed additional useful information, only the ever- versus never-vaccinated results are presented here.

Relative risks (RR) and 95% confidence intervals (CI) were calculated by conditional logistic regression (Breslow and Day, 1980) using a matched-pairs design and adjusting for age (in years) at censoring, year of birth, sex, race, family income and parental education, as shown in Table 1. For nine subjects (seven cases and two controls) with unknown family income, the median family income among controls was imputed as the subject's family income. For the fathers of 31 subjects (12 cases and 19 controls) whose level of education was unknown, the median education level from the controls was imputed. Since early day-care or preschool attendance has been linked with decreased risk of ALL in prior studies (Petridou et al, 1993), we adjusted for these variables as well.

Monovalent injected vaccines to protect against hepatitis B virus, tetanus and poliovirus were received by fewer than 1% of subjects, and monovalent vaccines to protect subjects from *Neisseria meningitidis*, *Mycobacterium tuberculosis*, varicella, or mumps, were received by very few subjects. Therefore, none of these vaccines were separately analysed. Although few subjects received monovalent or bivalent vaccines against tetanus and/or diphtheria, summary variables were created to integrate all possible vaccine doses containing tetanus or diphtheria toxoids.

The first Hib vaccine approved by the US Food and Drug Administration was a polysaccharide vaccine licensed in April 1985. A single dose was to be administered at the age of 24 months (Advisory Committee on Immunization Practices, 1985), or at 18 months for those in day-care, since early day-care attendance was linked with increased risk of invasive Hib infection in several US studies (Redmond and Pichichero, 1984; Istre et al, 1985; Cochi et al, 1986). The first conjugate vaccine was licensed in December 1987; a single dose was to be administered at the age of 18 months (Advisory Committee on Immunization Practices, 1988). The first doses of conjugate vaccine were shipped in 1988 (Adams et al, 1993). In 1990, two conjugate Hib vaccines, developed subsequent to the first formulation, were recommended for use in four doses at the ages of 2, 4, 6 and 15 months (Advisory Committee on Immunization Practices, 1991).

Since the vaccine records provided by mothers or physicians did not specify the type of Hib vaccine, any dose administered before 1 January 1988 was assumed to be polysaccharide Hib vaccine for purposes of this study; doses administered on or after that date were assumed to be the conjugate Hib vaccine. Only 29 subjects (14 cases and 15 controls) received more than one dose of Hib vaccine; 28 of these were born after 1988 and presumably received only conjugate vaccine. Due to small numbers, it was deemed impossible to assess the effect of multiple doses of Hib vaccine. No subject received Hib vaccine doses both before and after 1 January 1988. For the analysis of Hib vaccine effects, the polysaccharide and conjugate vaccine types were modelled simultaneously, and the reference group included only those subjects who received neither type.

### RESULTS

Demographic data on cases and controls (Table 1) show that cases came from families with lower income and parental education than controls, but there were few differences in year of birth, age at censoring, or attendance at day-care or preschool. Results for individual vaccines, as well as for the two summary variables reflecting all exposures to tetanus or diphtheria toxoids, are shown in Table 2. The vast majority of subjects received at least one dose of three major vaccines: oral poliovirus (98%), diphtheria–tetanus–pertussis (97%), and measles–mumps–rubella

 © Cancer Research Campaign 1999

**Table 2** Effect of vaccination (ever vs never) on subsequent risk of childhood acute lymphoblastic leukaemia (439 matched pairs)

| Vaccine | Cases | Controls | RR[a] | 95% CI[b] |
|---|---|---|---|---|
| Measles–mumps–rubella | 395 (90%) | 394 (90%) | 1.19 | (0.67–2.10) |
| Oral poliovirus | 429 (98%) | 428 (97%) | 1.05 | (0.41–2.67) |
| Diphtheria–tetanus–pertussis | 424 (97%) | 428 (97%) | 0.66 | (0.27–1.65) |
| Tetanus (all) | 431 (98%) | 430 (98%) | 0.75 | (0.26–2.16) |
| Diphtheria (all) | 431 (98%) | 430 (98%) | 0.75 | (0.26–2.16) |
| *Haemophilus influenzae* b (Hib) | 206 (47%) | 232 (53%) | 0.73 | (0.50–1.06) |
| (Presumptive) polysaccharide vaccine | 53 (12%) | 50 (11%) | 1.13 | (0.64–1.98) |
| (Presumptive) conjugate vaccine | 153 (35%) | 182 (41%) | 0.57 | (0.36–0.89) |

[a]Relative risk, adjusted for age at censoring, year of birth, sex, race, family income, parental education and attendance at day-care and/or preschool.
[b]Ninety-five per cent confidence interval.

(90%). None of these vaccines significantly altered the risk of subsequent ALL.

Hib vaccine in some form was received by 53% of controls versus 47% of cases (RR = 0.73; 95% CI 0.50–1.06). Similar proportions of cases (12%) and controls (11%) received the polysaccharide Hib vaccine (RR = 1.13; 95% CI 0.64–1.98). A higher proportion of controls (41%) than cases (35%) received the conjugate Hib vaccine; thus, the conjugate Hib vaccine was associated with a significantly reduced risk of subsequent ALL (RR = 0.57; 95% CI 0.36–0.89). When the effect of Hib vaccination was analysed for three different birth cohorts (pre-1984, 1984–1986, and post-1986), the reduced risk was seen only among children born after 1986, who presumably received only the conjugate Hib vaccine (data not shown). Neither age at vaccination nor interval between vaccination and diagnosis affected the risk of ALL following any of the vaccines; nor did number of doses of OPV or DTP (data not shown).

### DISCUSSION

Several vaccines commonly administered to US infants have been reported to reduce the risk of subsequent childhood leukaemia in four of the five previously-cited studies. Our study is one of the largest and most comprehensive evaluations of the relationship between infant vaccinations and childhood ALL. It is also distinguished by reliance on written vaccination records, rather than maternal recall. In addition, our study is one of the first to investigate the relationship between Hib vaccines and childhood ALL. Administration of the conjugate Hib vaccine was associated with a reduced risk of subsequent childhood ALL. The polysaccharide Hib vaccine exhibited no such inverse association. In the conjugate vaccine, the Hib capsular polysaccharide is covalently bound (i.e. 'conjugated') to a highly immunogenic protein antigen, yielding an antibody response which begins earlier (at a younger age), rises higher, and lasts longer than that which follows vaccination with the unconjugated polysaccharide (Wenger et al, 1989). Although the mechanism of any anti-leukaemic effect of vaccination remains to be elucidated, it is at least plausible that such an effect would be more pronounced with the conjugate vaccine.

In contrast with four previous studies (Kneale et al, 1986; McKinney et al, 1987; Nishi and Miyake, 1989; Kaatsch et al, 1996), our data provide no evidence of a reduced ALL risk following vaccination with DTP, MMR, or OPV. Our failure to detect such inverse associations may be due to a lack of statistical power, because DTP, MMR and OPV were nearly universal among both cases and controls. There are other possible reasons for this discrepancy, however. Our study differed in several methodologic aspects from previous investigations. Only one previous study (Innis, 1965) obtained vaccine data from written records, only one (Kneale et al, 1986) included more cases, and only two (Nishi and Miyake, 1989; Kaatsch et al, 1996) focused specifically on ALL. While four of the previous studies (Kneale et al, 1986; McKinney et al, 1987; Nishi and Miyake, 1989; Kaatsch et al, 1996) utilized age-matched controls, only one (Kaatsch et al, 1996) adjusted for socioeconomic status. Only one previous study (Nishi and Miyake, 1989) separately reported the relative risks and 95% confidence intervals for childhood leukaemia following specific vaccinations; other studies reported the effect of specific vaccines on all malignancies combined (Kneale et al, 1986), and/or the effect of vaccines in general on childhood leukaemia in particular (Innis, 1965; Kneale et al, 1986; McKinney et al, 1987; Kaatsch et al, 1996).

The apparent reduced risk of ALL after receiving conjugate Hib vaccine may have been a chance finding, perhaps due to multiple comparisons. Another possible explanation of the results for Hib vaccine may be bias, since controls selected using random-digit dialing were more likely to be offspring of parents with higher education and/or family income compared with cases (Kleinerman et al, 1997). While this may explain the tendency of the controls to have more vaccinations, the association between conjugate Hib vaccine and ALL persisted after statistical adjustment for family income and parental education, and no such inverse association was observed for any other vaccine. Confounding by socioeconomic status may be more plausible for the Hib vaccine than for other vaccines, since parents of higher socioeconomic status may have been more inclined to avail themselves of the newly-introduced, voluntary Hib vaccine.

Because both age and calendar time are strongly related to Hib vaccination rate, we controlled for both temporal factors. Although we assumed that only the conjugate Hib vaccine was administered after 31 December 1987, the polysaccharide vaccine might have been used sporadically. However, such misclassification would have reduced the observed inverse association. Other important limitations of our study were the use of random-digit dialing to identify controls, and the attendant low response rate (only 50%) for controls. The response rate was lower than that for other components of the study because written vaccination records with dates of vaccination were required; nonetheless, because of the requirement for written records, this is one of the largest and most stringent investigations to date of the role of vaccinations in childhood ALL.

© Cancer Research Campaign 1999

We believe that our findings justify a more definitive investigation of this question because of the important scientific and public health implications if our findings for the Hib vaccine are confirmed. Future studies should utilize population-based cases and controls matched as closely as possible on date of birth, with vaccination histories obtained from medical records. Cohort studies comparing the incidence of leukaemia among subjects who participated in randomized clinical trials of the conjugate Hib vaccine, or other vaccines, would offer stronger evidence to support or refute a reduced ALL risk following vaccination.

### ACKNOWLEDGEMENTS

The authors thank Ms Stella Semiti of Information Management Sciences, and Ms Shelley Niwa of Westat, Inc., both of Rockville, Maryland, for their valuable computer support, and Ms Carol Haines of Westat, Inc. for overseeing the data collection.

### REFERENCES

Adams WG, Deaver KA, Cochi SL, Plikaytis BD, Zell ER, Broome CV and Wenger JD (1993) Decline of childhood *Haemophilus influenzae* type b (Hib) disease in the Hib vaccine era. *J Am Med Assoc* **269**: 221–226

Advisory Committee on Immunization Practices (1985) Polysaccharide vaccine for prevention of *Haemophilus influenzae* type b disease. *Morbid Mortal Weekly Report* **34**: 201–205

Advisory Committee on Immunization Practices (1988) Update: Prevention of *Haemophilus influenzae* type b disease. *Morbid Mortal Weekly Report* **37**: 13–16

Advisory Committee on Immunization Practices (1991) *Haemophilus* b conjugate vaccines for prevention of *Haemophilus influenzae* type b disease among infants and children two months of age and older. *Morbid Mortal Weekly Report* **40 (RR-1)**: 1–7

Breslow NE and Day NE (1980) *Statistical Methods in Cancer Research. Vol. I. The Analysis of Case-Control Studies*, pp. 122–279. (IARC Scientific Publication No. 32.) International Agency for Research on Cancer: Lyon, France

Cochi SL, Fleming DW, Hightower AW, Limpakarnjanarat K, Facklam RR, Smith JD, Sikes RR and Broome CV (1986) Primary invasive *Haemophilus influenzae* type b disease: a population-based assessment of risk factors. *J Pediatr* **108**: 887–896

Innis MD (1965) Immunisation and childhood leukaemia. *Lancet* **1**: 605

Istre GR, Conner JS, Broome CV, Hightower A, and Hopkins RS (1985) Risk factors for primary invasive *Haemophilus influenzae* disease: increased risk from day-care attendance and school-aged household members. *J Pediatr* **106**: 190–195

Kaatsch P, Kaletsch U, Krummenauer F, Meinert R, Miesner A, Haaf G and Michaelis J (1996) Case-control study on childhood leukemia in Lower Saxony, Germany. Basic considerations, methodology, and summary of results. *Klin Padiatr* **208**: 179–185

Kleinerman RA, Linet MS, Hatch EE, Wacholder S, Tarone RE, Severson RK, Kaune WT, Friedman DR, Haines CM, Muirhead CR, Boice JD and Robison LL (1997) Magnetic field exposure assessment in a case-control study of childhood leukemia. *Epidemiology* **8**: 575–583

Kneale GW, Stewart A and Kinnier Wilson LM (1986) Immunizations against infectious diseases and childhood cancers. *Cancer Immunol Immunother* **21**: 129–132

McKinney PA, Cartwright RA, Saiu JMT, Stiller CA, Draper GJ, Hartley AL, Hopton PA, Birch JM, Waterhouse JAH and Johnston HE (1987) The inter-regional epidemiological study of childhood cancer (IRESCC): a case-control study of aetiological factors in leukaemia and lymphoma. *Arch Dis Child* **62**: 279–287

Nishi M and Miyake H (1989) A case-control study of non-T-cell acute lymphoblastic leukaemia of children in Hokkaido, Japan. *J Epidemiol Commun Health* **43**: 352–355

Petridou E, Kassimos D, Kalmanti M, Kosmidis H, Haidas S, Flytzani V, Tong D and Trichopoulos D (1993) Age of exposure to infections and risk of childhood leukaemia. *Br Med J* **301**: 774

Redmond SR and Pichichero ME (1984) *Haemophilus influenzae* type b disease: an epidemiologic study with special reference to day-care centers. *J Am Med Assoc* **252**: 2581–2584

Robison LL and Daigle A (1984) Control selection using random digit dialing for cases of childhood cancer. *Am J Epidemiol* **120**: 164–166

Wenger JD, Ward JI and Broome CV (1989) Prevention of *Haemophilus influenzae* type b disease: vaccines and passive prophylaxis. In: *Current Clinical Topics in Infectious Disease*, Remington JS and Swartz MN (eds), pp. 318–320. Blackwell Scientific Publishers: Boston

 © Cancer Research Campaign 1999
